# Hypoxia and activation of hypoxia inducible factor alpha as influencers of inflammatory helper T cells in autoimmune disease – a link between cancer and autoimmunity

**DOI:** 10.3389/fimmu.2025.1633845

**Published:** 2025-09-02

**Authors:** Giovanni Almanzar, Juan Carlos Alarcon, Ruth Garzon, Ana Maria Navarro, Alejandro Ondo-Méndez, Martina Prelog

**Affiliations:** ^1^ Department of Pediatrics, Pediatric Rheumatology/Special Immunology, University Hospital Würzburg, Würzburg, Germany; ^2^ Center for Proteomics and Metabolomics, Leiden University Medical Center (LUMC), Leiden, Netherlands; ^3^ Clinical Research Group, School of Medicine and Health Sciences, Universidad del Rosario, Bogotá, Colombia; ^4^ Facultad de Ciencias, Departamento de Química, Universidad Nacional de Colombia, Bogotá, Colombia; ^5^ Fundación Universitaria Ciencias de la Salud (FUCS), Servicio de Genética Médica, Bogotá, Colombia

**Keywords:** hypoxia, HIF-1α, helper T cells, cancer, autoimmunity

## Abstract

As a part of the tumor microenvironment, hypoxia is an important hallmark in the tumor progression. Hypoxia is a condition in which the oxygen supply is not sufficient to sustain the cell demand. In addition to its known impact in tumor progression, hypoxia seems to play a principal role in the generation and evolution of several autoimmune diseases. Both tumor and autoimmune diseases can be modulated by the hypoxia inducible factor alpha (HIF-1α) sharing similar molecular mechanisms. Here, we outline the links between cancer and autoimmunity regarding hypoxia-induced factors, such as HIF-1α, and describe the role of hypoxia in the modulation of the autoimmune response.

## Introduction

Clinically, hypoxia causes cellular stress resulting in inflammatory reactions leading to tissue and organ damage. From the cellular perspective, hypoxia triggers the activation of cell signaling pathways related to metabolism and inflammation trying to adapt to the new oxygen conditions (1). Autoimmune diseases are known as a broad spectrum of conditions in which humoral and cellular components of the adaptive immune system react to cells, matrix structures, and nuclear molecules. Autoimmune conditions are accompanied by inflammatory cytokines and induction of molecular signaling pathways, which lead to abundance of inflammatory helper, cytotoxic T cell types, and to dysfunctional regulatory mechanisms, e.g. mediated by regulatory T cells (Treg) or anti-inflammatory mediators (2). Additionally, B cells contribute also to tissue- and cell-destruction by production of autoantibodies (3). The etiopathogenesis of autoimmune inflammation is multifactorial and mostly based on genetic susceptibility which may be triggered by pathogen- and damage-associated molecular patterns (PAMPs and DAMPs, respectively) driven by microbial antigens and lead to the destruction of tissue or cell structures (4). Further, abundance of cellular and nuclear materials may fire the reactogenicity of the innate and adaptive humoral and cellular immune system. Consequently, hypoxia, which causes cell damage, activation of inflammatory mechanisms, and revealing cell and matrix surfaces may constitute a significant factor in the breakthrough of peripheral immune tolerance mechanisms, and, thus, set an individual at risk to develop autoimmunity and autoinflammation (5). The present narrative review aims to unveil links between hypoxia and autoimmune reactivity with particular emphasis on T cell-specific factors that highlights parallels to inflammatory mechanisms driven by tumor-induced hypoxia.

## Hypoxia pathway and hypoxia-inducible factor

Suitable oxygenation is essential for ensuring cell survival and appropriate cell function. Normal oxygenation of tissues lays in a narrow range, from 5 to 21 percent of oxygen, depending on the tissue and its metabolic requirements. For example, while there could be 21% of oxygen in lung tissue, tissues like the bone marrow are under lower oxygen tension (2 – 8% pO_2_) (6–9). The variability of oxygenation among tissues makes it difficult to establish a universal definition of hypoxia, so it has become more useful to define it in a functional way, in which hypoxia arises because of the imbalance of oxygen supply and consumption. In this situation, oxygen delivery does not meet the demands of the tissue resulting in anaerobic metabolism (10–12).

Tissue hypoxia can result from physiological factors such as physical activity and high altitude as well as from pathological factors, like pulmonary diseases, anemia, ischemia, chronic vascular disease, and cancer (13–16). Although hypoxia could be dangerous for every type of cell, metabolic and enzymatic plasticity provides the cells with the adequate mechanisms to adapt and survive under such harsh conditions. The metabolic shift is driven mainly by the activation of the hypoxia inducible factor (HIF) by increasing glucose uptake and the accumulation of metabolites such as lactate, citrate, and pyruvate, among others (17, 18). After the modulation of glycolytic enzymes such as hexokinase (HK) and phosphofructokinase (PKF) (19). This metabolic rewiring enables cells to generate ATP anaerobically under low levels of oxygen (20) and allows them to adapt and survive under hypoxic conditions. Another cell protecting mechanism activated by HIF are the activation of the superoxide dismutase (SOD) to neutralize the effect of reactive oxygen species (ROS) (21) and the increment in mitophagy to reduce mitochondrial damage under hypoxic conditions (22).

To maintain oxygen homeostasis, the human body senses oxygen concentration and responds to acute or chronic hypoxia. In sharp changes of oxygen, lasting seconds to minutes, changes in phosphorylation of cell signaling molecules and redox state in the cell are the principal response. Cells can switch their metabolism from aerobic to anaerobic energy generation through the increased expression of glucose transporters and glycolytic enzymes (18, 20, 21). Under chronic hypoxia, changes in gene expression in the cell are typical. The upregulation of HIF-1α and HIF-2α leads to transcriptional changes reprogramming metabolic processes switching to an inflammatory profile affecting macrophages and T cells (23, 24). Moreover, hypoxia affects macrophages polarization driving them to a proinflammatory phenotype M1-like (25). In the case of T cells, cytotoxic capacity can be impaired by a reduction in the IFNγ production (26), increasing regulatory T cell populations (27), and/or facilitating Th17 differentiation, all of them relevant for immune modulation. Tissue under this condition needs to reduce the total number of cells by the blocking cell cycle and cell proliferation, or by inducing apoptosis and necrosis as prominent cell death associated mechanisms (28, 29). Autophagy appears as a substitute mechanism when this adaptive response is not sufficient to overcome low oxygen conditions (30).

**Figure 1:**
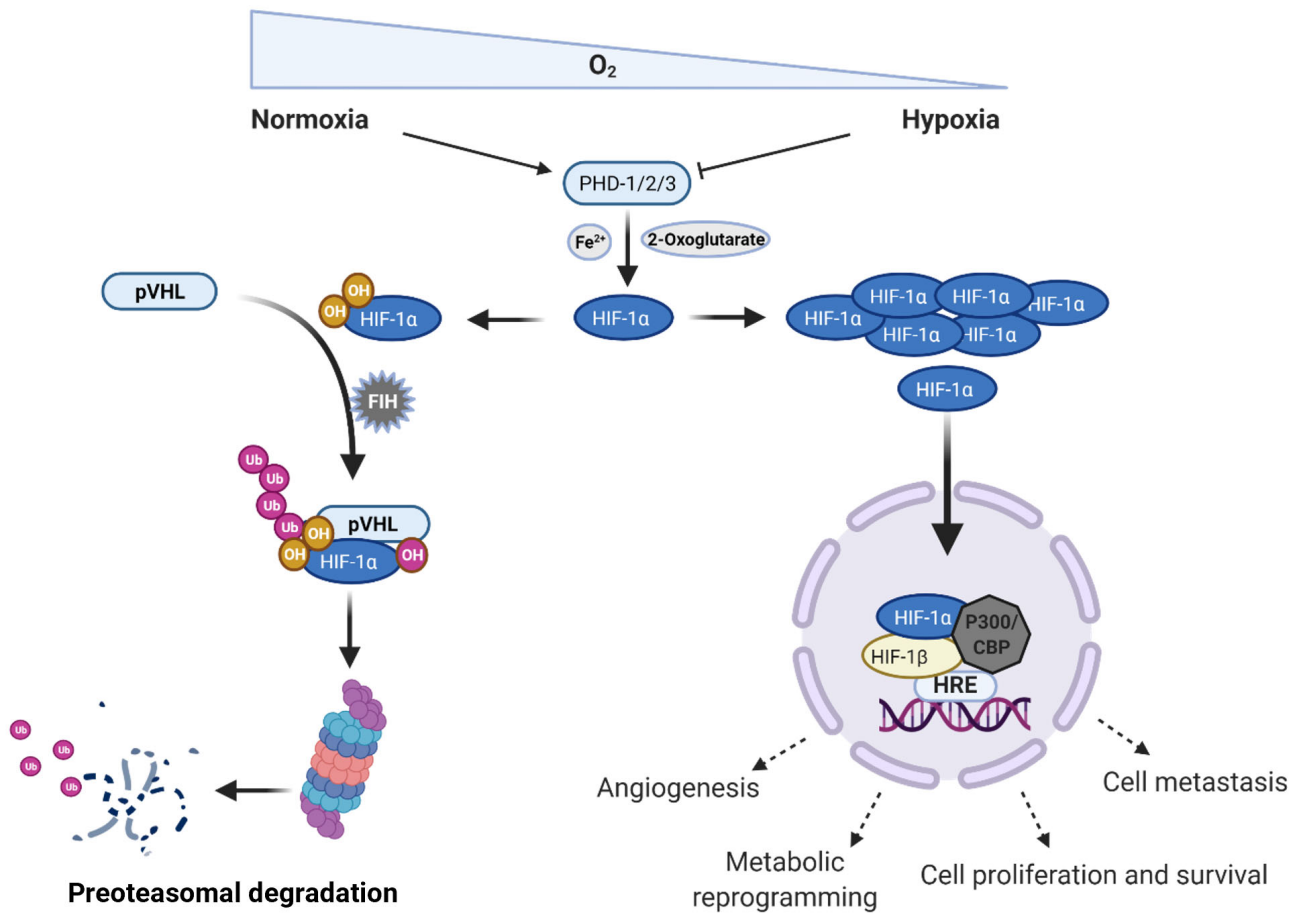
Regulation of the hypoxia inducible factor 1 alpha (HIF-1α) in normoxic and hypoxic conditions. Under normoxia, intracellular concentration of HIF-1α is regulated by different types of enzymes inducing its rapid proteasomal degradation; however, when the oxygen levels drop, HIF-1α can translocate to the nucleus and together with HIF-1β starts the transcription of genes related to different cell processes, such as angiogenesis, metabolic reprogramming, cell proliferation, and survival as well as metastasis. PHD, prolyl hydroxylases; pVHL, phosphorylated Von Hippel – Lindau tumor suppressor protein; FIH, factor inhibiting HIF-1 protein; HRE; hypoxia response elements; CBP, CREB-binding protein; Ub, Ubiquitin; OH, hydroxylation. Created in BioRender. Almanzar, G. (2025) https://BioRender.com/b1lf2y0.

The completely adaptive response is under control at the molecular level by the rapid accumulation and activation of HIF-1 (**Figure 1**). HIF-1 is a heterodimeric transcription factor constituted by two subunits, the regulatory hypoxia response factor HIF-1α, and the constitutive receptor nuclear translocator HIF-1β (31, 32). These subunits are members of a large family of transcription factors which share a basic helix-loop-helix region and a Per-Arnt-Sim (PAS) domain; these regions are necessary for the formation of the dimer, and to the recognition of specific DNA sequences to transcription factor binding in the nucleus (5´- XCGTG – 3´, X represents adenine or guanine) (33).

Low oxygen levels lead to accumulation of HIF-1α, which can translocate to the nucleus and form a dimer with the β subunit. This dimer interacts with the CH1 pocket of the transcriptional activator CBP/P300 and binds to hypoxia response elements (HREs), thereby activating the transcription of multiple target genes, such as phosphoglycerate kinase (*PGK1*), vascular endothelial growth factor A (*VEGFA*), glucose transporter (*GLUT1*), among others. Cellular processes that are regulated by HIF pathway include angiogenesis, glycolysis, growth-factor signaling, immortalization, genetic instability, tissue invasion and metastasis, apoptosis and pH regulation (34, 35).

There are two other isoforms for the regulatory subunit, HIF-2α and HIF-3α. The last one is less studied, but it has been described as an inhibitor of HIF-1α through a negative feedback that involves the inhibitory Per/Arnt/Sim domain protein (IPAS) promoter (36, 37). On the other side, HIF-2α has a similar structure to HIF-1α but it is only expressed in certain tissues (38). In addition to its tissue specific expression, right after dimerization with HIF-1β, HIF-2α promotes the activation of different genes principally related to tumor growth, cell proliferation and cell pluripotency (39).

Nevertheless, under non-hypoxic conditions, HIF-1α is degraded upon hydroxylation, and ubiquitylation. HIF-1α is hydroxylated on proline residues 402 and/or 564 by prolyl hydroxylases (PHD – 1/2/3), which are tetrameric enzymes with two hydroxylases subunits and two di-sulfide isomerase subunits (31, 35). These enzymes show high oxygen affinity and use oxygen as a co-substrate to introduce the hydroxylation in proline residues generating a 4 – hydroxyproline (40, 41). The hydroxyproline is recognized by the Von Hippel – Lindau (VHL) tumor suppressor protein that is the recognition component of an E3 ubiquitin-protein ligase complex. Thus, finally leads to the polyubiquitylation of HIF-1α and its proteasomal degradation by the 26S subunit. The prolyl hydroxylases also require a ferrous ion (Fe^2+^), a Krebs cycle intermediate (2 – oxoglutarate), and ascorbate as co-factor to reduce the Fe^3+^ (31, 35, 42). Transcriptional activity of HIF-1α can also be hampered by asparaginyl hydroxylase enzyme factor inhibiting HIF-1 (FIH), which disturbs its interaction with the CBP/P300 transcriptional activator (**Figure 1**) (35).

In conclusion, hypoxia response through HIF-1α is meant to increase the cell adaptive response to lower oxygen conditions by regulating energy and redox status, thereby promoting cell survival and proliferation. This mechanism is highly regulated in cells under normal oxygen levels; hence, its dysregulation conveys the appearance of many metabolic and cell deviations.

## Hypoxia and tumor microenvironment

Tumor microenvironment (TME) is the result of the paracrine and autocrine crosstalk of different cell types, controlling a variety of molecular processes with the aim to maintain and promote the tumor growth. It is considered as a region distinguished in space and function, which is comprised by different cell populations, namely, cancer cells, epithelial cells, extracellular matrix, stromal fibroblasts, immune cells (lymphocytes, macrophages, and mast cells), and vascular cells (endothelial, pericytes, and smooth muscle cells) (43, 44). One important feature of the TME is the presence of intermittent hypoxia which induces a higher glycolytic metabolism, resulting in extracellular acidosis; low nutrient availability, and probably failure of pH regulation (45). These specific conditions produce cellular stress, genetic variability, and up-regulation of genes such as the Eukaryotic translation initiation factor 4A, isoform 2 (*EIF4A2*), and Ribosomal protein L37 (*RPL37*) involved in supporting translation process, cell survival and invasion (46). In tumors, hypoxia is triggered by the up-regulation of cell proliferation that increases the oxygen requirements, which subsequently cause neovascular formation and cellular adaptation through metabolic and enzymatic adjustment (19, 47).

An interesting example comes from the 26S proteasome non-ATPase subunit 4 (*PSMD4*) gene, which encodes a ubiquitin-binding protein and constitutes one of the major ubiquitin receptor of 26S proteasome. A recent study showed that hypoxic conditions led to the upregulation of this gene through the direct binding of HF1 to the HRE in the *PSMD4* promoter (48). *PSMD4* overexpression correlated with poor survival rates in colon carcinoma and in breast cancer (49). Additionally, in hepatocellular carcinoma (HCC), the expression of *HF1A* and *PMSD4* showed to be highly correlated and both being strong indicators of the disease progression (50). Under hypoxia a metabolic shift from oxidative phosphorylation to glycolysis (the Warburg effect) takes place. HIF-1α induces upregulation of GLUT1 (SLC2A) facilitating glucose uptake across the plasma membrane, the hexokinase 2 (HK2) which phosphorylates glucose to glucose-6-phosphate, and conversion of pyruvate into lactate, regenerating NAD+ for glycolytic flux (51). However, to prevent intracellular acidification, cancer cells upregulate monocarboxylate transporter 4 (MCT4) to export lactate (52). Accumulation of lactate in the TME promotes angiogenesis, immune evasion, and matrix remodeling (53).

Hypoxic cancer cell survival is mediated by the overexpression of the anti-apoptotic Bcl-2 family member Mcl-1 in solid tumors, which is regulated by HIF-1α and correlates to poor survival (54). Additionally, HIF-1α activates BNIP3 inducing selective autophagy and reducing ROS (55). The carbonic anhydrase IX (CA9) plays an important role in regulating intracellular pH acting as buffer catalyzing the hydration of carbon dioxide (CO_2_) to bicarbonate (HCO_3_-) and a proton (H+) reducing the acidic environment created by lactate accumulation (56). The overexpression of CA9, specially in hypoxic tumor regions, is associated with metastasis and poor prognosis (57). The phosphoinositide 3-kinase (PI3K)/Akt signaling pathway is involved in hypoxia-ischemia and it is a major signaling pathway in various types of cancer controlling cell survival, metastasis, and metabolism increasing the production of insulin-like growth factor 2 (IGF2) (58). This activation drives phosphorylation of the PIK3 to generate phosphatidylinositol-3,4,5-trisphosphate (PIP3) through phosphorylation of phosphatidylinositol-4,5-bisphosphate (PIP2), recruiting Akt to the cell membrane, where it is activated by PDK1 (59). One of the downstream effectors of Akt, the mechanistic target of rapamicyn (mTOR) associates several other proteins involved in cancer progression (60). Thus, several components of the PI3K/Akt/mTOR pathway frequently show mutations and are activated in cancer (61, 62).

Hypoxic TME favors endothelial cell (EC) proliferation and migration. Deletion of p53 in cancer cells increases HIF-1α levels and enhances transcriptional activation of HIF-1α-dependent VEGF and erythropoietin (EPO) in response to hypoxia, thus promoting EC proliferation, migration, and angiogenesis (63, 64). Angiogenesis initiated from Tumor activated-endothelial cells (TECs), resulting in the production of vascular endothelial growth factor A (VEGF-A), angiopoietin-like 4 (ANGPTL4), placental growth factor (PIGF), and platelet-derived growth factor B (PDGF-B), all of them, contribute to the endothelial cell proliferation, migration, and capillary formation. However, the hypoxia-angiogenesis cycle occurs because the newly generated vasculature is usually immature, leaky, and poorly perfused, which reduces the oxygen supply and reinforces hypoxia (65–67).

TECs maintain close contact with circulating immune cells, both innate and adaptive, and mediates their interactions with tumor stroma, thus, TECs play a pivotal role as sentinels and immune regulators performing tissue- and vessel-type-specific immunomodulatory functions, including recruitment, activation and antigen presentation of immune cells. Activated ECs recruit effector immune cells guiding their infiltration into the tumor microenvironment through a multi-staged adhesion process which includes binding of the integrins lymphocyte function-associated antigen-1 (LFA-1) and very late antigen-4 (VLA-4) on T cells to the respective ligands intercellular adhesion molecule-1 (ICAM-1) and vascular cell adhesion protein-1 (VCAM-1) on TECs (68). The tumor-derived cytokines such as vascular endothelial growth factor (VEGF), endothelin-1 (ET1), EGF-like domain-containing protein 7 (EGFL7), and fibroblast growth factor 2 (FGF2) downregulate gene expression and protein expression of adhesion molecules and chemoattractants (e.g., CCL2, CXCL10, and CXCL7) resulting into inhibition of the immune cell infiltration (69).

Cancer associated fibroblasts (CAFs), an important component of the tumor stroma in many types of cancer such as colorectal and breast cancer, illustrate another example of the role of hypoxia in the tumor progression. Although CAF cells can be derived from multiple cells types (resident fibroblast, endothelial, epithelia, mesenchymal stem cells, pericytes, adipocytes) they lack the protein expression for endothelial, epithelial on hematopoietic cells, but they do express mesenchymal biomarkers such as vimentin or platelet derived growth factor receptor alpha (PDGFR-α) (70). These cells can be regulated by both HIF-dependent and HIF-independent mechanisms (71). Under hypoxic conditions, activated CAFs cells can mediate cancer progression by activating pathways that regulate extracellular matrix remodeling, immune response, metabolic reprogramming, angiogenesis and metastasis (72).

Interleukin-6 (IL-6) is the predominantly cytokine released by CAFs in response to the activation of the signal transducer and activator of transcription 3 (STAT3) via HIF-1α. This downstream cascade target PKM2 and facilitates the expansion of CAFs (70). Furthermore, hypoxia increases the mRNA and protein expression of anti-tumor immunity factors including TGF-β, IL-10, VEGF, CXCL12, PD-L1, FasL, and CD39 on CAFs. Therefore, hypoxia improves the immunosuppressive function of CAFs in the TME (71).

In addition to promoting the hypoxia dependent CAF expansion, IL-6 is also produced in the early stage of the TME development, it is strongly associated with the proinflammatory response during infection and immune cell activation. IL-6 is important for the differentiation of naïve CD4+ T cells into IL-17-producing T helper (Th17) cells in association with TGF-β (72). In contrast, IL-6 inhibits TGF-β-induced Treg differentiation (73) contributing to the dysbalance between Th17/Treg described in several T cell mediated autoimmune diseases (74–76) such as psoriasis, rheumatoid arthritis (RA), and inflammatory bowel diseases (IBD) (75–77). IL-6 exacerbates inflammation facilitating differentiation of CD4+ T cells into Th17, inducing tissue damage and supporting fibrosis by increasing the levels of extracellular matrix components (78, 79). High levels of IL-6 and the soluble IL-6 receptor alpha chain (IL-6Rα) play an important role in suppression of the immune response in tumor progression of non-small cell lung cancer (NSCLC) (80). IL-6 has been reported to induce and maintain the cancer stem cell state (81, 82). IL-6 can be secreted by resident macrophages (83), fibroblasts upon activation (84), or by the presence of T cells during cellular response (85). Thus, data regarding the key cytokine IL-6 suggests a link between the role of hypoxia in tumor progression and the mechanisms used by hypoxia in the progression of inflammation and autoimmunity.

## Immune evasion in the hypoxic tumor microenvironment

**Figure 2:**
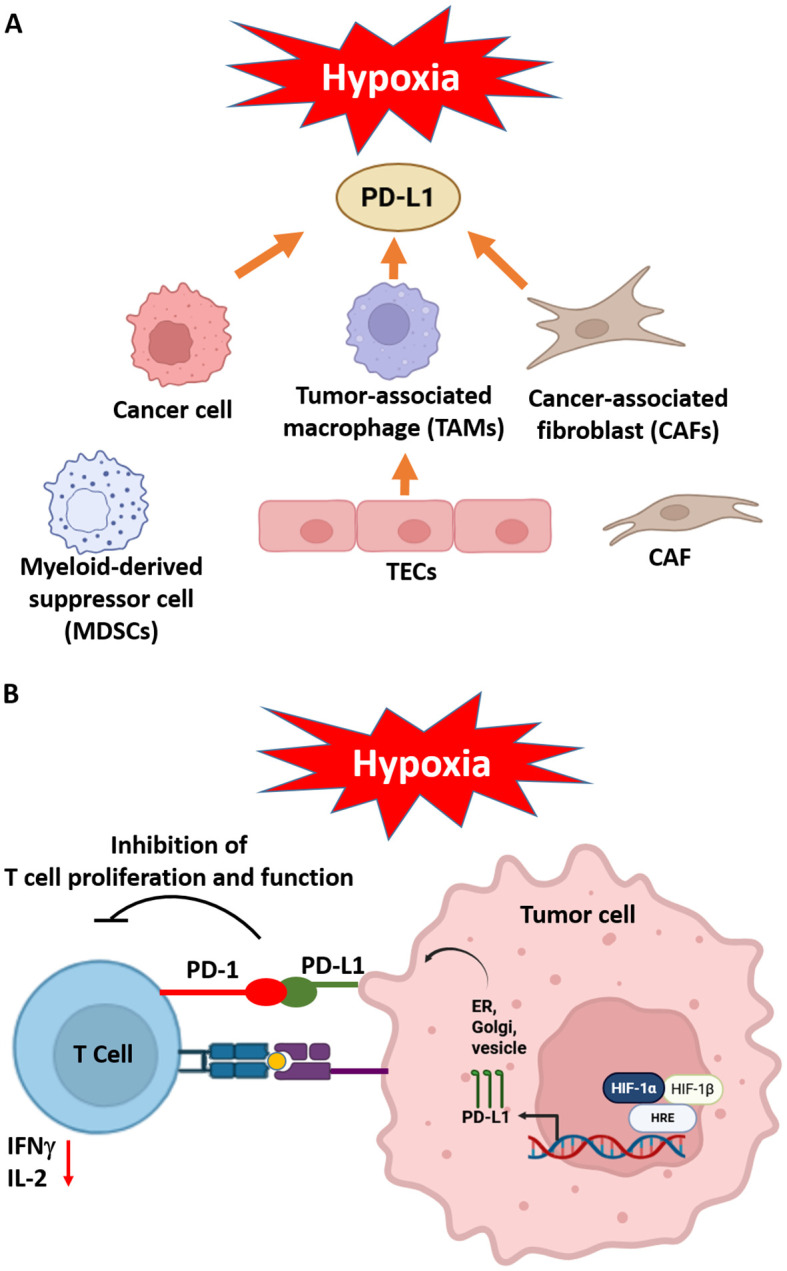
Hypoxia generates an immunosuppressive environment inducing PD-L1 expression in tumor microenvironment components such as cancer cells, tumor-associated macrophages (TAMs), cancer-associated fibroblasts (CAFs), myeloid-derived suppressor cells (MDSCs) and tumor endothelial cells (TEC) **(A)** Hypoxia increased levels of HIF-1α binds to HRE in the PD-L1 promoter stimulates the PD-L1 production, which is transported to the surface of the tumor cell **(B)** The presence of PD-L1 on cells in the tumor microenvironment (TME) results in the binding of PD-1, which in turn inhibits the activity of tumor T cells reducing IFNγ and IL-2 levels and consequently leads to tumor progression. Adapted from (94). Created in BioRender. Almanzar, G. (2025) https://BioRender.com/b1lf2y0.

Hypoxia profoundly shapes the immunological landscape of the tumor microenvironment by promoting immune evasion through multiple mechanisms. A central feature of this process is the HIF-1α–mediated upregulation of immune checkpoint ligands, particularly programmed death-ligand 1 (PD-L1), on tumor cells and associated stromal cells (86). PD-L1 binding to its receptor PD-1 on cytotoxic CD8^+^ T cells leading to T cell exhaustion and diminished antitumor immunity (87, 88). HIF-1α binds directly to HRE in the promoter region of the PD-L1 gene, enhancing the transcriptional upregulation of the ligand in several tumor types such as lung, breast, renal, head and neck cancers (86). PD-L1 expressed on cancer cells binds to PD-1 on activated CD8+ T cells inhibiting T cell proliferation, reducing IFNγ and IL-2 production, and cytolytic activity (89, 90). As consequence, features of T cell exhaustion are identified and enable tumor cells to resist immune-mediated elimination. PD-L1 expression under hypoxia is induced on tumor-associated macrophages (TAMs), myeloid-derived suppressor cells (MDSCs), CAFs, and endothelial cells creating an immune-privileged niche. PD-L1+ TAMs bind to PD-1 on NK cells impairing their cytotoxic function (91). MDSCs are recruited to hypoxic tumor areas via CCL2 and CXCL12, express PD-L1 and suppress antigen presenting cells function affecting DC maturation and impairing T cell priming (92). CAFs remodel the extracellular matrix and express PD-L1 inhibiting T cell proliferation (93), while PD-L1+ endothelial cells suppress trans-endothelial migration of T cells blocking T cell activation at tumor entry. In fact, PD-L1 expression induced by hypoxia acts as a gatekeeper mechanism allowing Treg or MDSCs to enter the tumor while excluding CD8+ cytotoxic T cells (**Figure 2**).

In parallel, hypoxic tumor cells secrete a repertoire of immunosuppressive factors, including transforming growth factor-beta (TGFβ), interleukin-10 (IL-10), and vascular endothelial growth factor (VEGF) (95). These soluble mediators not only suppress the activity of effector T cells and NK cells but also assist the recruitment and expansion of immunosuppressive populations such as Treg and MDSCs. Collectively, these hypoxia-driven alterations establish a profoundly immunosuppressive microenvironment that supports tumor-associated immune escape and limits the efficacy of immunotherapeutic interventions.

## Regulatory T cells in the hypoxic microenvironment

**Figure 3:**
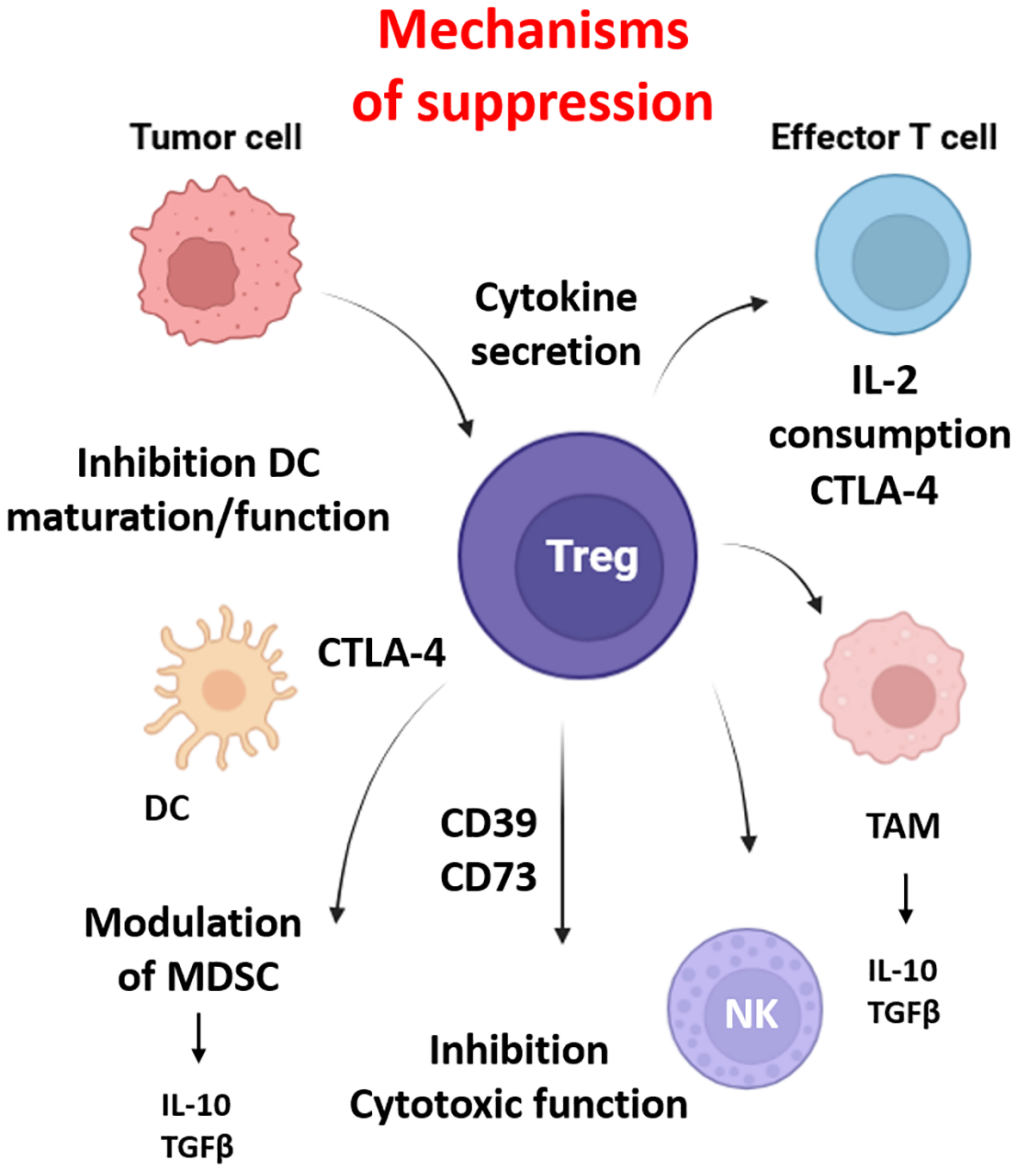
Regulatory T cell (Treg) mediated immunosuppression and cellular interactions in the tumor microenvironment. Regulatory T cells suppress anti-tumor immunity within the tumor microenvironment (TME). Tregs inhibit CD8^+^ cytotoxic T lymphocytes via CTLA-4–mediated competition for co-stimulation, IL-2 consumption, and secretion of suppressive cytokines (IL-10, TGF-β), which are produced by myeloid-derived suppressor cells (MDSCs) and tumor-associated macrophage (TAMs). They impair dendritic cell (DC) maturation and antigen presentation, convert extracellular ATP to immunosuppressive adenosine through CD39/CD73, and can directly kill effector cells via granzyme B/perforin. Tregs also interact with tumor-associated macrophages (TAMs), promoting M2-like polarization and reinforcing the immunosuppressive niche. Together, these actions contribute to immune evasion, therapy resistance, and tumor progression in hypoxic and immunosuppressive regions of solid tumors. Created in BioRender. Almanzar, G. (2025) https://BioRender.com/b1lf2y0.

Treg are characterized by the high expression of the IL-2 Rα (CD25) and the transcription factor forkhead-box-protein 3 (FoxP3) in CD4+ T cells, which are responsible for immune tolerance and homeostasis. However, the TME actively promotes Treg recruitment, stability, and suppressive function increasing resistance to anti-tumor immunity (**Figure 3**).

In hypoxic tumors, HIF-1α enhances the expression of chemokines such as CCL20, CCL22, CCL28, and CXCL12 establishing a chemotactic gradient that selectively recruit CCR6-, CCR10- and CCR4-expressing Treg (96). Treg in hypoxia also upregulate CTLA-4, IL-10, and TGFβ, which increase suppressive activity on effector T cells, NK, and DCs. CTLA-4 binds to CD80/CD86 on DCs downregulating co-stimulatory molecules and inducing indoleamine 2,3-dioxygenase (IDO) depleting tryptophan and stopping T cell proliferation. IL-10 and TGFβ are produced by TAMs and MDSCs contributing to the Treg differentiation (97, 98). Additionally, the expression of CD39 and CD73 on Treg generate adenosine from extracellular ATP. Adenosine interacts through the G-coupled purinergic type 1 receptor such as A2A receptors on effector cells and NK cells to inhibit cytotoxic function and cytokine production such as IFNγ and IL-2 (99, 100). IL-2 consumption via CD25 also contributes to reduce the effect of conventional T cells (101).

## HIF and inflammation

Inflammation describes a clinical phenomenon related to increased flow in local blood vessels, increased cellular infiltration, and changes in tissue metabolism and oxygen supply. The inflammatory microenvironment is characterized not only by low molecular weights mediators, such as lipid mediators, cytokines, and chemokines secreted by different cell types (leucocytes and monocytes) but also by low levels in oxygen and nutrients (102–104). In acute and extensive inflammatory conditions, oxygen supply is decreased by poor tissue perfusion due to the microvasculature disruption e.g. by edema, vasculitis or vasoconstriction as found in many autoimmune conditions (105–107).

During an inflammatory condition, hypoxia and many other mechanisms from the immune response can induce a stable expression of HIF-1α. For example, during inflammation cytokines like IL-1β, IL-6 and TNFα increase the translation of the mRNA of HIF-1α in a NF-κB dependent way, through the activation of the PI3K/AKT/mTOR pathway (102, 108). Another mechanism involves the bacterial lipopolysaccharides (LPS), which can bind to the cell surface receptors CD14 and TLR-4 stimulating the p44/MAPK/ERK1/2 pathway leading to the activation of the transcription factor NF-κB, and the up-regulation of HIF-1α mRNA (102, 109). The inflammatory condition is also associated with perturbations in oxidative stress and redox cell state. For example, it was reported that IL-1β, IL-6, IL-8, and TNFα promote the activation, stabilization, and translocation of HIF-1α in a ROS depending way (110). One more example is the nitric oxide (NO) produced by macrophages and granulocytes, which are important players of the innate immune system during inflammation. NO has been shown to stabilize HIF-1α by two mechanisms, the first one, by attenuating HIF-1α ubiquitylation and reducing its degradation; the second one is related with the inhibition of PHD by sequestering of Fe(II) ion co-factor (102, 111).

HIF-1α has been associated with the regulation of metabolism, vascularization, and matrix remodeling. During inflammation for instance, HIF-1α promotes the transcription of genes like *VEGF, SLC2A1* (encodes GLUT1) and those related to metalloproteinases (112–114). In macrophages and neutrophils, HIF-1α has been defined as the main regulator of glycolytic energy production. Impaired aggregation and motility have been observed in mice with deletion of myeloid HIF-1α (115). The HIF-1α – NFκB pathway is also involved in stimulating macrophages and reducing apoptosis in neutrophils through proinflammatory cytokine release (114, 116). Protein expression of FIH, prolyl hydroxylase domain enzymes 1 (PHD1) and 2 (PHD2) are not affected by hypoxia, whereas up-regulation of PHD3 has been observed in neutrophils under hypoxic conditions or isolated from rheumatoid patients (117, 118).

Macrophages and neutrophilic granulocytes also express HIF-2α in response to hypoxia. While HIF-1α is essential for survival, HIF-2α stimulates prevalence in the course of sterile inflammation in neutrophils (117).

## Th17 and Treg cell regulation and HIF

**Figure 4:**
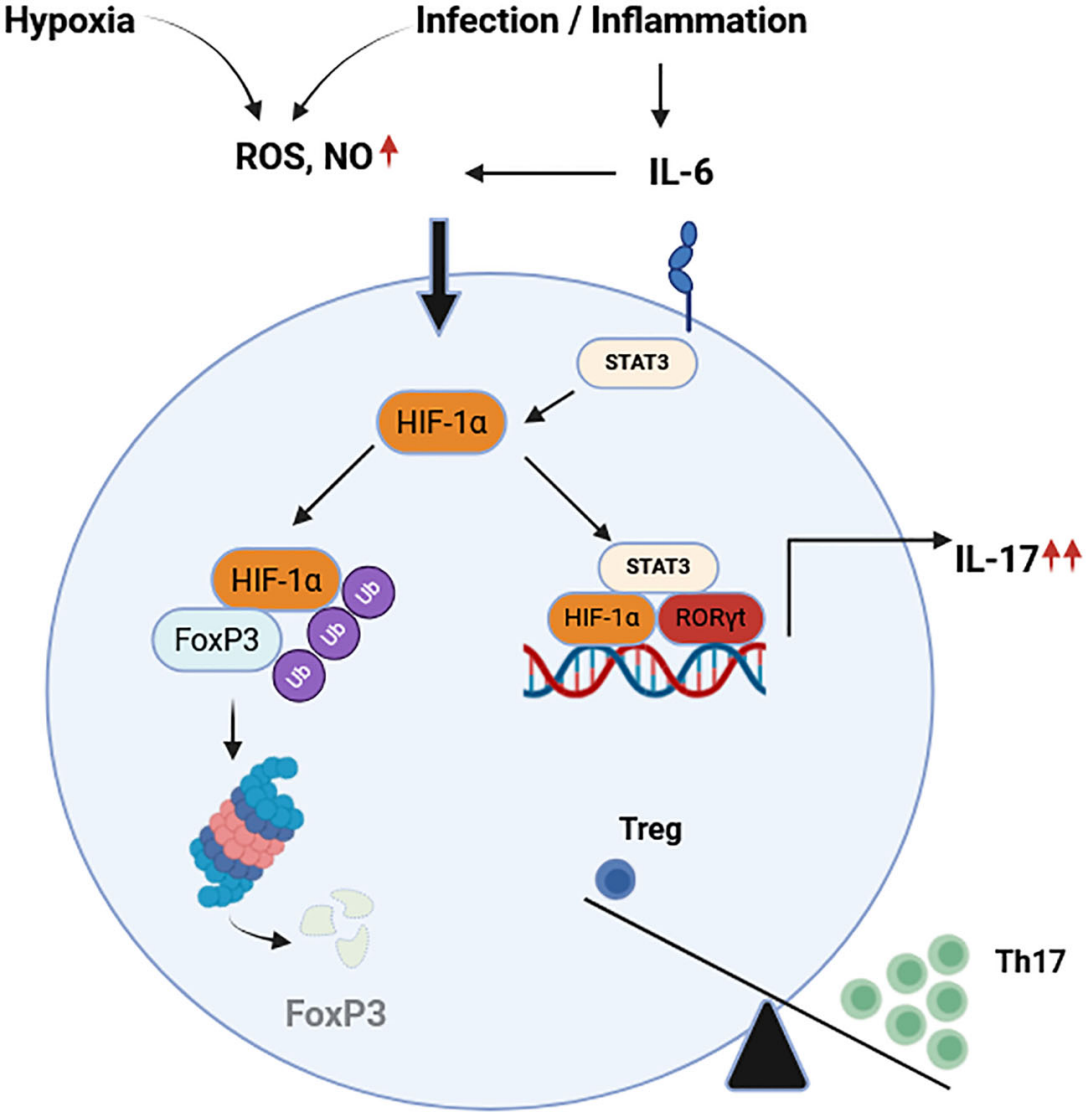
Hypoxia and infection or inflammation control the balance of Th17/Treg. Hypoxia and inflammatory conditions increase the levels of ROS, NO, and pro-inflammatory cytokines such as IL-6. On one hand, HIF-1α promotes the degradation of FoxP3 in proteasome regulating the differentiation of Treg. On the other hand, HIF-1α and STAT3 enhance expression of RORγt driving transcription of the Th17 genes and protein expression. These factors contribute to the imbalance between Th17 and Treg described in several T cell mediated autoimmune diseases. HIF-1α, hypoxia inducible factor 1 alpha; FoxP3, Forkhead-Box-Protein 3; STAT3, signal transducer and activator of transcription 3; ROS, reactive oxygen species; NO, nitric oxide; RORγt, retinoid-related orphan receptor gamma t; IL-6. Interleukin 6; IL-17, interleukin 17; Th17, T-helper 17 cells; Treg, regulatory T cells. Adapted from (119). Created in BioRender. Almanzar, G. (2025) https://BioRender.com/b1lf2y0.

Treg are essential for maintaining immune tolerance and preventing autoimmunity, but in the context of cancer, their immunosuppressive function becomes detrimental by enabling tumor immune evasion. A balance that is intricately regulated alongside Th17 cell differentiation by HIFs within the tumor microenvironment. HIF-1α regulates inflammation responses by modulating the activation and differentiation of Th17 and Treg cells in several manners (**Figure 4**).

This regulation involves a metabolic shift stemmed from the cell fate rewiring and the boost in cell expansion (120). For example, compared with other cell populations (Th1, Th2 or Treg), Th17 differentiation requires up-regulation in the glycolytic pathway in a HIF-1α dependent manner (121). During this process, both HIF-1α translation and transcription increased under independent pathways (122). To date, two pathways have been described, the first involves T cell receptor (TCR) and phosphoinositol-3-phosphate/mammalian target of rapamycin PI3K/mTOR pathway, in which the TCR can also induce the expression of splicing isoforms of HIF-1α mRNA whose function remains still unclear (123, 124). The second mechanism includes IL-6 and STAT3 pathway, both of which are associated in the differentiation of naïve T cells into Th17 cells. Although the molecular regulation and machinery involved are still unknown, many studies report a STAT3 mediated Th17 differentiation which also involves the HIF-1α induction in a hypoxia independent manner (24, 122).

Once HIF-1α is up-regulated in T cells, it readily associates with the promoter of the retinoid-orphan receptor gamma t (RORγt) which is necessary to stabilize the Th17 polarization. The combination of gene reporters and co-immunoprecipitation has shown that HIF-1α not only promotes RORγt gene expression, but also promotes the differentiation into Th17 cells (122). Moreover, HIF-1α regulates the expression of FoxP3 under inflammatory conditions (119). Studies including stimulation of HIF-1α knockout T cells showed no changes in the levels of FoxP3 mRNA in wild type (WT) and HIF-1 α^-/-^ T cells stimulated under Th17 conditions. However, FoxP3 protein expression was diminished in this condition. Additionally, induction of Treg in WT and HIF-1 α^-/-^ T cells upon *in vitro* treatment with IL-2 and TGFβ showed a reduction in the expression of the FoxP3 on the knockout cells compared to WT. Thus, HIF-1α down-regulates FoxP3 protein expression not affect the FoxP3 mRNA levels, suggest a mechanism that promotes the association of HIF-1α and FoxP3 and their further degradation via ubiquitination in the proteasome (119). This observation has been supported by other studies in which the absence of HIF-1α in T cells resulted in an up-regulation of FoxP3 and hence modulating cell metabolism in favor of lipid oxidation and down-regulating glycolytic metabolism (120).

The Th17 cell population can be derived from naïve cells by blocking the Aryl Hydrocarbon Receptor (AhR) (87). The AhR is regulated via STAT3, through the JAK/STAT pathway and the upregulation of IL-27. AhR and STAT3 are important transcription factors up-stream of RORγt for polarization of naïve T cells into the Th17 lineage, contribute to the membrane expression of CD39 enzyme. Through this enzyme the Treg population reduces the extracellular ATP and hence to the regulation of inflammation. Extracellular ATP is important for the differentiation of naïve T cells into Th17 cells (125, 126). Under hypoxic conditions, HIF-1α induces ubiquitination and degradation of AhR in proteasomes resulting in differentiation into regulatory type 1 T cells (Tr1). Tr1 produce IL-10 and play an important role in suppress autoimmune reactions (127). In Rheumatoid arthritis (RA) patients have shown negative correlations between expression of HIF-1α in Treg and disease activity score 28 (DAS28) (128). In contrast, the upregulation of HIF-1α promotes Th17 cell differentiation and hence to progression of the disease. Upregulation of HIF-1α has been associated with many other autoimmune and autoinflammatory conditions. For instance, HIF-1α over-expression in colon biopsies of patients with active IBD has been associated to increased expression of the macrophage inflammatory protein 3alpha (MIP-3α), and the presence of high serum levels of VEGF (129). Accumulation of HIF-1α has been identified in persistent pathofibrogenesis and may play a role in progression of systemic sclerosis (SSc), an inflammatory autoimmune disease resulting in abnormal fibroblast activation and fibrosis leading to skin thickening and organ insufficiency. HIF-1α promotes the deposition of extracellular matrix (ECM) and, thus, increases fibrosis (130). High levels of circulating Th17 cells and IL-17-producing “Th17-like” Treg have been identified previously in SSc patients. Functional assays demonstrated an impaired suppressive capacity of Treg cells that may be associated with the progression of the disease (76). Therefore, hypoxia may play a multifactor role in the progression of the SSc disease contributing to tissue remodeling, expansion of Th17 cells, and plasticity of Treg towards inflammatory helper T cell types.

## Tumor-induced HIF-1α, Th17, and pathogenesis of autoimmunity

**Table 1** summarized some features of the effect of hypoxia on tumor development and autoimmunity. Reduced levels of oxygen may induce a high rate of cancer cells proliferation, generating areas where hypoxia can spread heterogeneously within the tumor mass, especially in solid tumors (132). It has been estimated that 50-60% of tumors contain hypoxic regions, which have been associated with cancer metastasis and resistance to chemo and radiotherapy, suggesting hypoxia as indicator of poor prognosis in many types of cancer such as hepatocellular carcinoma, breast, pancreas, gastric and colorectal (133). In particular, it has been observed that adaptation in metabolite-driven gene regulation can be a powerful hallmark for measuring tumorigenesis (134).

**Table 1:** Regulation of Treg and Th17 cells by hypoxia in cancer and autoimmunity.

Context	Cell Type	Effect of Hypoxia (via HIF-1α)	Functional Outcome	References
Cancer	Treg	• HIF-1α promotes FOXP3 stability and enhances the suppressive function of Treg. • Upregulates PD-L1, CD39/CD73 on Treg	Enhances immunosuppression, facilitating tumor immune evasion	(27, 131)
	Th17	• HIF-1α promotes RORγt expression and Th17 differentiation. • May support tumor-promoting inflammation	Contributes to chronic inflammation and tumor progression	(119)
Autoimmunity	Treg	• Hypoxia may destabilize FOXP3 in inflammatory environments. • Impaired suppressive function of Treg	Reduces immune tolerance, exacerbating autoimmune responses	(119, 121)
	Th17	• HIF-1α strongly favors Th17 over Treg lineage commitment. • Increases IL-17 production	Promotes pro-inflammatory responses and tissue damage in autoimmune diseases	(119, 121)

Under cancer conditions, hypoxic levels in the cellular microenvironment inhibited the normal physiological processes, leading to stable accumulation of HIF-1α and HIF-2α (134). As a result, the HIF-1 complex can bind to HRE and recruit their coactivators p300 and the cAMP-response element binding protein (CREB)-binding protein to activate the transcription of multiple genes that respond to hypoxia (133). Namely, VEGFA, endocrine-gland-derived VEGF (EGVEGF), insulin-like growth factor-2 (IGF2), P53 and P21, transforming growth factor-β (TGF-β), SLC2A1, lactate dehydrogenase-A (LDHA), matrix metalloproteinases (MMPs), and nitric oxide synthase (NOS2) (132).

HIF-1α and HIF-1β are primary oxygen-limiting sensors and their induction support cancer cell proliferation during hypoxia and diverse metabolic alterations. HIF-2 α is sensitive to the availability of oxygen in tumors and promotes tumor progression through the lactate axis of macrophages (134).

Consequently, the TME particularly under hypoxic conditions significantly reprograms glucose metabolism to favor the pentose phosphate pathway (PPP) over oxidative phosphorylation, a phenomenon often associated with the Warburg effect (135, 136). This metabolic shift is primarily driven by hypoxia-inducible factor-1 alpha (HIF-1α) (137, 138),. HIF-1α upregulates the expression of glycolytic enzymes and glucose transporters, increasing glucose uptake and its diversion into the PPP (139). Therefore, part of the glucose-6-phospahte is diverted to generate nicotinamide adenine dinucleotide phosphate (NADPH), which neutralized ROS by glutathione reductase and thioredoxin systems maintaining redox balance (140, 141). NADPH is vital for maintaining cellular redox homeostasis by reducing oxidative stress, a common challenge in rapidly proliferating cancer cells, thus protecting them from ROS-mediated damage and apoptosis (135, 136). Additionally, the PPP supplies ribose-5-phosphate which server as a precursor for nucleotide biosynthesis, supporting the high proliferative demand of cancer cells and contributing to genomic instability (135, 142). This metabolic adaptation provides cancer cells with a significant advantage, enabling sustained proliferation, evasion of immune surveillance, and the production of growth factors that stimulate angiogenesis and ultimately facilitate metastasis (143–146). Therefore, the deviation of glucose to the PPP, orchestrated by factors like HIF-1α in the TME, is a critical mechanism by which cancer cells acquire hallmarks of malignancy, making HIF-1α and PPP enzymes promising targets for therapeutic intervention (137, 138, 147–149).

In activated immune cells, such as macrophages, dendritic cells, and T cells, NADPH promotes anabolic growth and the generation of ROS via NADPH oxidase for process such as microbial killing and immune signaling (150–152). However, dysregulated PPP flux in macrophages and T cells alters redox balance and promotes inflammatory pathways, skewing toward Th17 responses and impairing clearance of apoptotic debris (153) and on the other hand, G6PD-deficient immune cells exhibit poor oxidative burst and impaired bacterial killing, highlighting NADPH’s dual role in both ROS production and antioxidant defense (136).

Dysregulation of PPP activity has been implicated in the pathogenesis of autoimmune diseases, particularly systemic lupus erythematosus (SLE) (154). In SLE, elevated PPP flux has been observed in both T cells and monocytes. This contributes to increased NADPH production and ROS generation, exacerbating oxidative stress and promoting aberrant immune activation (155, 156). For example, the hyperactivation of the PPP in CD4^+^ T cells from patients with SLE correlates with increased mTOR signaling, T cell survival, and pro-inflammatory cytokine production (157–160). Furthermore, excessive NADPH supports lipid synthesis and nucleotide biosynthesis, thereby facilitating the proliferation and survival of autoreactive lymphocytes (161). These metabolic alterations amplify inflammatory responses and impair regulatory mechanisms, thereby sustaining autoimmunity (162–164). Preclinical studies have shown that targeting key enzymes in the PPP, such as glucose-6-phosphate dehydrogenase (G6PD), can reduce oxidative stress and restore immune balance (165). In rheumatoid arthritis (RA), high levels of serum toll-like receptor 2 (TLR2) and increased TLR2 expression on CD4+ T cells have been observed. This is associated with TNFα secretion and increased production of ROS, as well as reprogramming of glucose metabolism via HK2, which promotes the PPP (166). This suggests that modulating the PPP may offer novel therapeutic strategies for SLE and related autoimmune conditions.

The PPP plays an important role in regulating Treg function. However, deletion of the enzyme transketolase (TKT) in mice induces impaired Treg suppressive function, despite normal FoxP3 expression levels and leads to unhampered oxidative phosphorylation causing fatal autoimmune disease (167).

Hypoxia may also contribute to tumor escape from the immune system affecting the number and functionality of the natural killer cells (NK), T cells, and dendritic cells (DCs) by regulating the expression of HIF-1α (168). Additionally, in some autoimmune diseases such as rheumatoid arthritis, diabetes mellitus type 1, multiple sclerosis and intestinal inflammatory disease, among others, high concentrations of HIF-1α have been reported, which correlates with a higher activity of the disease (169). Thus, HIF-1α is not only involved in tumor progression and escape from immune surveillance but may also directly affect T cell functionality.

Increased levels of IFNγ have been shown in activated T cells under hypoxia (170). Reduced levels of oxygen and presence of an inflammation cytokine milieu contribute to the tissue damage (171, 172), and stimulate IFNγ production in effector cells under Th17-driving conditions, suggesting a switch towards Th1 responses (1).

Low oxygen levels stabilize HIF-1α disrupting mitochondrial function and increasing ROS impairing metabolic fitness in activated T cells (173). These metabolic stresses trigger intrinsic apoptotic pathways, characterized by mitochondrial outer membrane permeabilization, cytochrome C release, and activation of caspase-9 and caspase-3 (174–176). Hypoxia also enhances the expression of pro-apoptotic molecules such as Bcl-2-interacting mediator of cell death (BIM) and the p53 upregulated modulator of apoptosis (PUMA), while downregulating survival factors like Bcl-2 (177). Moreover, tumor and stromal cells in hypoxic regions upregulate PD-L1, Fas ligand (FasL), and galectin-9, which engage death receptors on T cells (e.g., PD-1, Fas, TIM-3) to initiate extrinsic apoptosis (89, 178, 179).

Previously, Sun et al. demonstrated that T cells undergoing apoptosis under hypoxic conditions showed an inhibition in the expression of chemokine C receptor 7 (CCR7) (180). T cells lose their homing migration capacity to regional secondary lymphatic organs reducing tumor-specific antigen-presentation. Therefore, increased T cell apoptosis probably impairs the antitumor T cell response resulting in tumor progression (181).

Rheumatoid arthritis (RA) is a chronic inflammatory disease characterized by lymphocyte tissue infiltration, cytokine release, and joint damage (182). High levels of HIF-1α in serum and increased number of HIF-1α expressing cells have been detected in synovial tissue from RA patients (183). Under hypoxic conditions and high levels of IL-17, HIF-1α promotes synovial angiogenesis by increasing VEGF expression and exacerbating migration of fibroblast-like synovial cells through enzymatic activity of the metalloproteases 2 and 9 (MMP2 and MMP9) escalating bone destruction (184). Moreover, high levels of HIF-1α in fibroblast-like synovial cells stimulate expansion of Th1 and Th17 cells and increase concentrations of IFNγ and IL-17 (185).

Psoriasis arthritis (PsA) is characterized by inflammation of synovial tissues with hyperplasia of the joint surface, enhanced vascularity, and presence of inflammatory cells (186). Serum levels of VEGF and HIF-1α are significantly elevated in PsA patients compared to healthy controls or PsA patients in remission (187) suggesting a direct effect of the HIF-1α factor in the increased expression of VEGF which induces proliferation, migration, and differentiation of endothelial cells (188, 189).

Inflammatory bowel diseases (IBD) are chronical intestinal inflammatory diseases characterized by dysfunction of the intestinal barrier and tissue hypoxia (190). HIF-1α stimulates production of IL-1β inducing conversion of macrophages into the M1 phenotype (191). The M1 phenotype is characterized by high levels of inducible nitric oxide synthase (iNOS) (192), expression of HLA-DR and CCR7 (193). M1 macrophages secrete proinflammatory cytokines such as TNFα, IL-6 and iNOS promoting colitis (194, 195). HIF-1α is abnormally secreted in inflamed tissue from Crohn’s disease activating VEGF signaling promoting angiogenesis. Supporting this observation, HIF-1α^-/-^ mice showed weight loss and displayed severe intestinal inflammation suggesting a protective role of HIF-1α in inflammatory mechanisms involved in the immune regulation of IBD (196). Taken together, HIF1-α may display a protective and anti-pathogenic role in autoimmune colitis.

## Epigenetic changes by hypoxia in inflammation

Tumor infiltrating lymphocytes have a predominantly Th17 phenotype due to the presence of high levels of Th17-polarizing cytokine in the tumor microenvironment described in several cancer types (197, 198). IL-6 and the signal transducer and activator transcription 3 (STAT3) upregulate the histone motif H3K4me3 on the *IL-17* locus. Epigenetic regulators of the tripartite motif-containing 28 (TRIM28) are activated through TCR signaling and together with H3K4me3 permissive histone mark allow binding of the RORγt leading to the production of IL-17 (199). Th17 cells can be recruited to the tumor sites by the expression of CCR6 and binding to its natural ligand CCL20 highly expressed on tumor tissues (197, 200), which is coordinated by long non-coding RNA (lncRNA-u50535) (197). Interestingly, CCR6 and CXCR3-Th17/Treg cells secreting IL-17 have been described in colorectal and esophageal cancer, while the presence of IL-17-producing-FoxP3+ cells is related with poor prognosis because they have been shown to promote tumor progression (201, 202). Th17 plasticity is associated with epigenetic modifications. Mark of H3K27me3 to the *IL17* and *IL21* loci in Treg correlated to permissive marks of H3K4me3 in Th17 cells (203). Taking together, hypoxia may compromise epigenetic modifications that accelerate and explain conversion of Th0 into Th17 cells promoting tumor proliferation and impairing T cell response to tumor antigens.

## The critical role of hypoxia in cancer therapy

Understanding the molecular and biochemical mechanisms of tumor hypoxia is key to improving cancer treatments. Recognition of hypoxia as a major factor in treatment resistance dates back to the 1950s. Gray et al. (1953) (204) showed that hypoxic tissues require much higher radiation doses than oxygenated ones, identifying hypoxia as a cause of radio resistance. This finding highlighted the challenge of oxygen deficiency in solid tumors. Later research confirmed that hypoxia promotes tumor growth, aggressiveness, and resistance to therapies like chemotherapy and radiotherapy (205, 206).

Hypoxia drives immune evasion, abnormal angiogenesis, and metabolic shifts, leading to poor treatment response. In the context of radiotherapy, treatment efficacy depends on the generation of reactive oxygen species (ROS) to induce DNA damage in cancer cells. Hypoxic conditions, characterized by low oxygen levels, directly compromise this mechanism, as oxygen is essential for fixing radiation-induced DNA damage. This results in radio resistance, requiring higher radiation doses to achieve tumor control (207). The hypoxic microenvironment also promotes autophagy contributing to radio resistance by enabling cancer cells to survive under stress conditions (208). Studies using nasopharyngeal carcinoma xenografts have shown that hypoxia reduces radiation sensitivity, and overcoming this requires strategies that suppress the repair of DNA double-strand breaks under hypoxic conditions (209). Imaging techniques such as the ^18^F-fluoromisonidazole (^18^F- FMISO PET) have been developed to localize hypoxic regions within tumors, offering the potential to guide adaptive radiotherapy and escalate radiation doses to resistant areas (210, 211). However, clinical implementation of these approaches faces challenges related to cost, time, and accessibility (211).

Chemotherapeutic agents—particularly those whose mechanisms of action depend on oxygen—are significantly impacted by tumor hypoxia. Hypoxia can reduce drug uptake, alter metabolism, and induce cellular adaptations that confer resistance. For example, mitomycin C (MMC) treatment in ovarian cancer cells under hypoxic conditions exhibits extracellular matrix (ECM) modifications rather than ribosome-related pathways currently observed under normoxia (212). The efficacy of cisplatin (CisPt) in ovarian cancer cells is diminished under hypoxia, and even natural compounds like resveratrol, often used as adjuvants, lose their cytotoxic effects in low-oxygen environments (213).

Immunotherapy using checkpoint inhibitors (ICIs) (e.g., anti-PD-1, anti-PD-L1, anti-CTLA-4) is limited by hypoxia, which upregulates immunosuppressive molecules and attracts regulatory T cells and suppressor cells (214, 215). Therefore, immunotherapy is more effective in tumors with high immune cell infiltration. Thus, use of anti-angiogenic drugs such as bevacizumab or anlotinib may reduce hypoxia through vascular normalization to enhance infiltration of immune cells in tumors. Therapeutic approaches including use of ICIs and anti-hypoxia may represent a promising strategy to reduce tumor progression (215). The tumor microenvironment, particularly hypoxic regions, plays a major role in modulating immune responses, frequently leading to immunosuppression and resistance to immunotherapies. Hypoxia reinforces immunosuppresion inhibiting cytotoxic T cell and NK cell function (206). However, the use of hypoxia-activated prodrug evofosfamide has shown promise in enhancing breast cancer cell sensitivity to apoptosis and NK cell-mediated cytotoxicity under hypoxic conditions, in part by preserving type I interferon signaling, which is otherwise suppressed in hypoxia (216). Modulation of TME by targeting hypoxia may enhance effectiveness of immunotherapy (217).

Nanoparticle-based delivery systems offer an alternative therapeutic approach to specifically targeting hypoxic tumor cells. These nanoparticles can deliver anti-hypoxic agents directly to the TME improving oxygenation and boosting the effectiveness of conventional therapies (218). In hepatocellular carcinoma (HCC), the combination of HIF-1α inhibitors with metabolic modulators such as palmitic acid and L-carnitine has shown potential in inducing apoptosis in hypoxic HCC cells by preventing lipid metabolism reprogramming (138). Interestingly, is the possibility to combine nanoparticles carrying metabolic inhibitors with ICIs to increase T cell function diminishing T cell exhaustion (219).

In the era of genetic and molecular medicine, gene-editing technologies such as CRISPR-Cas9 aim to permanently eliminate the HIF-1α gene (220), thereby reducing tumor growth and enhancing T cell-mediated immunity. RNA interference therapies using siRNA or shrank can silence HIF-1α mRNA, preventing its translation and reducing hypoxia-driven angiogenesis and immunosuppression (221).

## Conclusions

**Figure 5:**
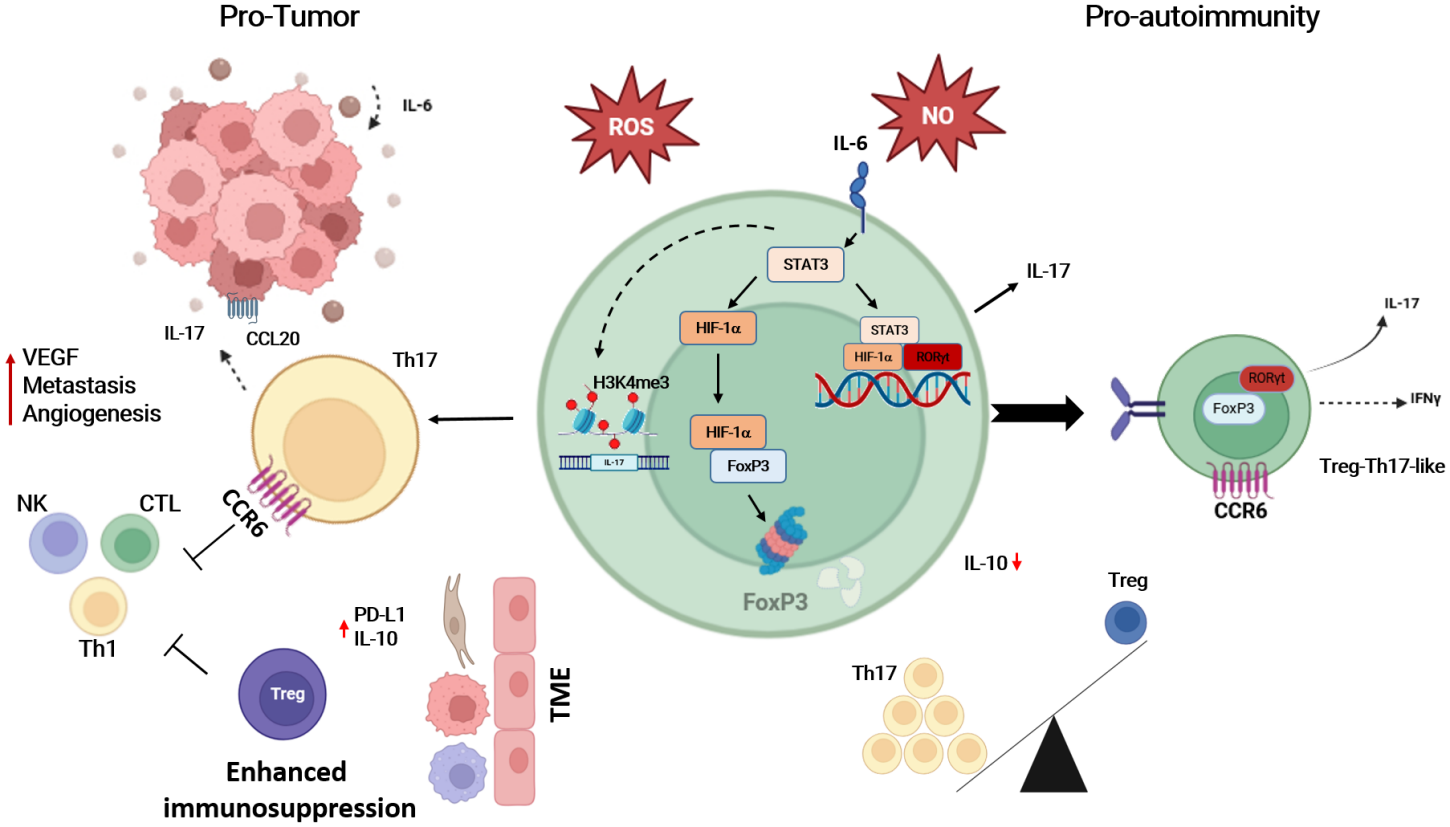
The up-regulation of HIF-1α under hypoxic conditions is likely to be the key to T cell differentiation into Th17 cells, which contributes to tumor progression and/or the triggering of autoimmune diseases. The abundance of IL-17 is not only a product of STAT3 induction, but hypoxia also drives the recruitment of Th17 cells to the tumor microenvironment (TME). This blocks the anti-tumor effect of NK, Th1 cells, and CTLs, and is also responsible for the impaired function of Treg, either by degrading FoxP3 or by enhancing Treg plasticity towards Treg-Th17-like T cells. On the other hand, TME increased expression of PD-L1 and IL-10 recruiting Treg and enhancing immunosuppression. Th17, T-helper-17 cells; Treg, regulatory T cells; NK, natural killer cell; Th1, T-helper-1 cells; CTL, cytotoxic T cells; IL-17, interleukin-17; IL-6, interleukin-6; IL-10, interleukin-10; IFNγ, interferon-gamma; CCR6, chemokine receptor 6; CCL20, chemokine ligand 20; HIF-1α, hypoxia inducible factor 1 alpha; FoxP3, Forkhead-Box-Protein 3; STAT3, signal transducer and activator of transcription 3; RORγt, retinoid-related orphan receptor gamma t; ROS, reactive oxygen species; NO, nitric oxide. Created in BioRender. Almanzar, G. (2025) https://BioRender.com/b1lf2y0.

Inflammatory and tumor microenvironments are similar regarding the composition of cells and features involved in both pathogenetic processes (222). Increased and stable expression of HIF-1α due to a reduction of the oxygen concentrations is a common feature observed in both pathologic conditions, tumor progression and (auto)inflammation. HIF-1α translocates into the nucleus and binds to HRE to initiate transcription of target genes involved in angiogenesis, invasion/metastasis, and chemoresistance (223). On the other hand, HIF-1α is associated with plasticity of Th1, Th17 and Treg cells toward Th17-like phenotype, suggesting that HIF-1α plays an important role in tumor progression and T cell immune response modulation by induction of inflammatory T cell phenotypes. The presence of Th17 polarizing cytokines such as IL-6 in inflamed microenvironment and the upregulation of HIF-1α contribute to the functional imbalance between pro-inflammatory T cells and Treg cells. In addition, hypoxia may support plasticity of Treg cells in detriment of their regulatory function on one side accelerating the degradation of the FoxP3 in the proteasome and enhancing proportions of Treg with a Th17 –like phenotype described also in several autoimmune diseases (75, 224). Thus, the evidence suggests a common mechanism in tumor progression and autoimmune disease development involving the regulation of HIF-1α, which contributes to the differentiation of T cells into pro-inflammatory cells characteristic of many autoimmune diseases (**Figure 5**).

Due to immunological dysregulation, and in part also fired by immunosuppressive therapy in autoimmune disorders, cancer rates are higher in those patients with autoimmune conditions compared to the general population (225–227). HIF-1α and other hypoxia-induced mechanisms may be the missing link to understand the increased risk of tumorigenesis in those patients suffering from autoimmune diseases. Unraveling the molecular and epigenetic pathways that govern HIF-1α expression in T cell regulation under inflammatory and tumor-promoting conditions is essential to develop targeted therapies to interrupt the signaling pathways towards hypoxia-induced inflammation promoting tumor progression or manifestation of autoimmunity. Therefore, it is mandatory to gain better insights to define common hypoxia-associated hallmarks for (auto)inflammation and cancer that may further be used for the development of new therapeutic treatments.
